# What Effect Does International Migration Have on the Nutritional Status and Child Care Practices of Children Left Behind?

**DOI:** 10.3390/ijerph13020218

**Published:** 2016-02-15

**Authors:** Renuka Jayatissa, Kolitha Wickramage

**Affiliations:** 1 Department of Nutrition, Medical Research Institute, Ministry of Health, Colombo 00800, Sri Lanka; 2 Migration Health Division, International Organization for Migration (IOM), Headquarters, Geneva 1200, Switzerland; wickramage.kolitha@gmail.com

**Keywords:** labour migration, migrant families, nutritional status, children left behind, child care

## Abstract

Despite an increasing trend in labour migration and economic dependence on foreign migrant workers in Sri Lanka, very little is known about the child care and nutritional status of “children left behind”. The aim of this study was to examine the factors influencing the nutritional status and care practices of children left behind. A sample of 321 children, 6–59 months old of international migrant workers from a cross-sectional nationally represented study were included. Care practices were assessed using ten caregiving behaviours on personal hygiene, feeding, and use of health services. Results revealed the prevalence of stunting, wasting and underweight to be 11.6, 18.2 and 24.0 percent, respectively. Father being a migrant worker has a positive effect on childcare practices and birthweight of the child. This study indicates that undernutrition remains a major concern, particularly in the poorest households where the mother is a migrant worker, also each additional 100 g increase in the birthweight of a child in a migrant household, decreases the probability of being wasted, stunted and underweight by 6%, 8% and 23% respectively. In depth study is needed to understand how labour migration affects household level outcomes related to child nutrition and childcare in order to build skills and capacities of migrant families.

## 1. Introduction

International labor migration is a critical determinant of economic development for many low to middle income countries. It also plays a part in transforming traditional roles of parenting and caregiving practices for millions of “children left behind” by migrant workers [1]. Though migration is important within the global development agenda, health and migration policies recognising family interest and child health outcomes have not yet been adopted, especially in low to middle income countries [2]. The critical need for global health researchers to harness greater empirical evidence on the health status of migrants and mobile populations has also been highlighted [3,4].

Understanding how parental migration affects household poverty, food security, child care and other welfare indicators which underlie child nutrition is vital. Exploring the effects of parental migration on malnutrition of children left-behind also becomes a key health policy issue for many nations of the world, which are major source countries for labour migrants. The United Nations Children Fund (UNICEF) conceptual framework identifies three layers of causal factors; immediate (diet and disease), underlying (food security, child care and healthy environment) and basic causes of child undernutrition [5]. Childcare forms an important, yet complex concept within this framework that includes a range of behaviors and practices of caregivers which provide food, healthcare, stimulation, and emotional support necessary for the child’s healthy survival, growth and development [6]. Parental migration and the remittances they send to families left behind influence the child through at least two broad pathways at the household level. Firstly, the increased household income may increase purchasing power of food, better health care and supplies for a cleaner environment (soap, clean water, *etc.*), which may result in better improvements in child nutrition. Secondly, by increasing the labour and household burdens of the remaining parent or caretaker thereby limiting his/her time to devote to childcare practices, *i.e.*, reducing the time spent to prepare food and/or to care for the child’s nutritional needs [7].

Labour migration continues to be an integral factor of Sri Lanka’s economic development with more people “on the move” than at any other time in recorded history. It is currently estimated that 1.7 million (8.5% of the total population) are working overseas and annually more than 200,000 persons are moving abroad for employment. The migrant workers remittances remain the highest foreign exchange earner to the national economy which is estimated to be 8% of Gross Domestic Products (GDP) [8]. Sri Lanka follows Asia’s labour migration trends which consist mainly of movement of workers to the Middle East—primarily to the Gulf Cooperation Council (GCC) countries, where many work in low-skilled jobs within precarious employment settings. The majority of migrant workers belong to the 25–29 age group, with 63.2% being male [8]. Many workers take continuous cycles of re-migration to increase savings and pay off recruitment fees of migration agents as well as other hidden costs of migration [9]. After adopting the National Migration Health Policy in 2014, the Government of Sri Lanka has committed to provide an enabling policy and a program platform to balance monetary gains from migrant worker remittances for poverty alleviation in order to ensure health, nutrition and social protection of migrant workers and their families [10]. To do so, the Ministry of Health has embarked on an evidence based process of policy and program planning.

In Sri Lanka, there is a persistent high prevalence of under nutrition among children, despite good indicators related to areas of maternal and child health [11]. However, there has been limited research on the topic of migration and the nutritional status of children left behind and caregiving practices within migrant households [12,13,14,15]. Hence the aim of this study was to examine the factors influencing the care practices and nutritional status of Sri Lankan children left behind when at least one parent migrates abroad for work.

## 2. Methods

### 2.1. Data Sources

Data of the Sri Lanka national nutrition and micronutrient survey were used for analysis [16]. This was a representative cross-sectional household survey conducted in 2012 and included 7500 children between 6–59 months. Sample size was calculated for this survey based on the 40% prevalence of micronutrient deficiency, to have a 95% confidence interval with a 5% margin of error considering a 10% non-response rate and a design effect of 1.5. The calculated sample size was 300 from each district. The smallest administrative unit in a district, “Gramasevaka area”, consisting of a population of approximately 1000, was taken as the Primary Sampling Units (PSUs). Thirty PSUs from each district were selected proportionate to the population. The starting point of each PSU was randomly identified using the map superimposing the numbered grid. Considering the household composition, 30–35 households were listed from that point and 10 randomly selected households with children 6–59 months in age were included in the survey. Food intake data was collected using un-weighted 24 h food recall questionnaire and 7 day semi-quantitative food frequency questionnaire using the standard methodology. The child’s mother or in the absence of the mother, the immediate caretaker was interviewed. Survey duration was August to December 2012.

In the survey, children’s weight and height were measured by standardized trained staff using UNICEF UNISCALES and stadiometers [17]. Weights and heights were taken to the nearest 0.1 kg and 0.1 cm respectively. Measures created were *height for age z-score (HAZ),* which measures long-term undernutrition, *weight for height z-score (WHZ),* which reflects acute undernutrition, and *weight for age z-score (WAZ),* which tends to assess both chronic and acute undernutrition [18]. The cut-off point for stunting, wasting and underweight was a standard deviation (SD) score (z-score) below −2SD of the reference value, according to World Health Organisation (WHO) guidelines [17]. All subjects gave their informed consent for inclusion before they participated in the study. The study was conducted in accordance with the Declaration of Helsinki, and the protocol was approved by the Ethics Committee of Medical Research Institute, Colombo (project 12/2010).

### 2.2. Measures of Child Caregiving Behaviour

Three kinds of caregiving practices, resulting in 10 separate behaviors were examined based on reported data considering the scoring system provided by Engle *et al.* [19], which has been used multiple times in other countries, based on personal hygiene (four variables), food and feeding (five variables), and use of health services (one variable).

The hygiene variables were whether the caregiver was always washing hands with soap, before cooking, before feeding the child, before eating and after using the toilet. These responses were collected under five categories (always with soap, sometimes with soap, without soap, do not wash and no answer). The five food and feeding variables were currently breastfeeding, usage of bottles, past 24 h dietary diversity, past 24 h frequency of feeding solids and semi-solid food, and past 7 days food frequency of feeding from different food groups [20].

The variable used for health care was giving Vitamin A supplementation during last 6 months [21]. A childcare practice score (a total of 10 point scale) was created using the scoring system defined by Ruel *et al.* [22]. It was used as a continuous variable for analysis.

### 2.3. Data Analysis

In the original dataset, age was calculated with birthdays extracted from the child’s health records or birth certificate that was available for all the children. Health status during the past 2 weeks was assessed by identifying the prevalence of diarrhea (three or more loose stools per day), acute respiratory tract infections (ARI-cough with or without fever) and fever (viral fever diagnosed by a doctor). Household wealth index was developed using housing characteristics, household possessions, availability of water and sanitation facilities. After which they were divided into five equal groups from the poorest to richest. Stunting, wasting and underweight were defined as height-for-age, weight-for-height and weight-for-age z-scores each being less than 2, using the WHO growth standards in AnthroPlus 2009 software [23]. Migrant households were defined as those in which one or both parents of the survey index child had migrated internationally for labour at the time of the survey, otherwise the household was considered as non-migrant.

Descriptive analysis by migrant households was examined. Explanatory variables used in the analysis included: household-level variables on maternal education; wealth; urban/rural residency; number of household members and child-level variables of sex, age, birth weight, care practices and health status (presence of diarrhoea, cough, fever) prior to two weeks. Poorest wealth quintile, urban residency, male, birthweight <2500 g, presence of diarrhoea, ARI and fever were considered as a value of 1.

Aim of this study was to estimate the likelihood of being a stunted/wasted/underweight child to changes in the explanatory variables, based on the migrant status and factors associated with this. The appropriate model to use would be the Probit model, which is a linear probability model with the binary dependent variable taking value 1 if the child is stunted and 0 otherwise. This same model was used to estimate the likelihood of being wasted or not; underweight or not.

The forward step-wise regression technique is used to select the significant variables to a child’s nutritional status adjusting for potential confounders based on UNICEF’s conceptual framework. Correlation matrix was prepared to identify the association of childcare practices with the explanatory variables.

## 3. Results

In the national dataset of children aged 6 to 59 months, 321 and 6985 belonged to migrant and non-migrant households respectively. In the migrant sample, 83.2% had a migrant father, 14.3% had a migrant mother and 2.5% had both parents working abroad. As shown in Table 1, migrant sample had older children (35.1 months *vs.* 32.8 months), more boys (53.3 percent *vs.* 50.1 percent), more living in urban locations (15 percent *vs.* 11.7 percent), more from the poorest and poor wealth quintiles (24 percent *vs.* 20.4 percent and 28.7 percent *vs.* 20.2 percent) than the non-migrant sample. The prevalence of stunting, wasting and underweight was lower in the migrant than non-migrant (11.5 percent *vs.* 14.8 percent, 18.1 percent *vs.* 21.5 percent and 24.3 percent *vs.* 26.2 percent respectively).

**Table 1:** Child and household characteristics for migrant and non-migrant households ^1^.

Child and Household Characteristics	Overall *N* = 7306	Migrant *N* = 321	Non-Migrant *N* = 6985
	**Mean (SD)/(%)**		
Nutritional status of children			
Stunting	11.5	11.5	14.8
Wasting	19.1	18.1	21.5
Underweight	23.1	24.3	26.2
HAZ	−0.9 (1.1)	−0.8 (1.2)	−0.9 (1.1)
WHZ	−1.2 (1.0)	−1.1 (1.1)	−1.2 (1.0)
WAZ	−1.3 (1.0)	−1.2 (1.1)	−1.3 (1.0)
Child’s factors			
Age of the child (months) ***	32.9 (14.7)	35.1 (14.2)	32.8 (14.8)
Child’s sex: boy	50.2	53.3	50.1
Birth weight (kg)	2.9 (1.3)	2.9 (0.5)	2.9 (1.3)
Child’s health status in prior 2 weeks			
Diarrhoea	7.5	5.9	7.6
Cough and cold	32.0	33.0	31.9
Fever	23.1	22.4	23.2
Maternal education ^1^			
No schooling	0.5	0.3	0.6
Primary school	3.5	7.6	5.0
Secondary school	19.4	60.1	66.9
High school	66.5	26.0	22.8
Beyond high school	5.3	5.9	4.6
Residency: urban	17.3	15.0	11.7
Wealth Index ***			
Poorest	20.0	24.0	20.4
Poor	20.0	28.7	20.2
Middle	21.0	20.6	19.2
Rich	20.0	13.1	20.5
Richest	19.0	13.7	19.6
Household members	4.4 (1.2)	4.4 (1.5)	4.4 (1.2)

^1.^ 33 missing: due to the absence of mother and caretaker not being aware of mother’s education); *** Significant at the 1% level.

As presented in Table 2, stunting and underweight is higher among children whose mother is a migrant worker. Households with only a migrant father were observed to have children with higher birthweight and younger children, while households with only a mother being a migrant worker were the poorest.

In this sample, there is a statistically significant negative association between child care practices and the younger age of a child as well as if the father is a migrant worker. In addition, there is a statistically significant positive association between childcare practices and HAZ, WAZ, birth weight, mother’s education and wealth index (Table 3).

**Table 2:** Association of child and household variables by the migrant status of the parents ^1^.

Child and Household Variables		Mean (SD)/(%)	
	Father Abroad	Mother Abroad	Both Abroad
Nutritional status of children			
Stunting	9.7	21.7	12.5
Wasting	18.0	17.4	25.0
Underweight	22.8	34.8	12.5
HAZ	−0.8 (1.2)	−1.1 (1.2)	−1.3 (1.5)
WHZ	−1.1 (1.0)	−1.3 (1.3)	−1.3 (1.5)
WAZ	−1.2 (1.0)	−1.1 (1.2)	−0.6 (0.1)
Child’s factors			
Age of the child (months) ***	33.4 (14.1)	44.5 (11.3)	36.3 (14.5)
Child’s sex: boy	55.1	43.5	50.0
Birth weight (kg) ***	2.9 (0.5)	2.7 (0.5)	2.5 (0.5)
Child’s health status in prior 2 weeks			
Diarrhoea	9.8	0.0	0.0
Cough and cold	26.3	28.3	62.5
Fever	23.7	17.4	50.0
Maternal education ^1^			
No schooling	0.0	2.6	0.0
Primary school	4.1	30.8	0.0
Secondary school	61.0	53.8	62.5
High school	28.6	10.3	25.0
Beyond high school	6.2	2.6	12.5
Residency: urban	16.5	8.7	0.0
Wealth Index			
Poorest	20.6	43.8	25.0
Poor	28.5	30.4	25.0
Middle	22.1	13.0	12.5
Rich	13.1	8.7	37.5
Richest	15.7	4.3	0.0
Household members	4.3 (1.4)	4.7(1.8)	5.1 (1.2)
Total	267 (83.6)	46 (16.4)	8 (2.5)

^1.^ 33 missing; *** Significant at the 1% level.

**Table 3:** Correlation of child and household variables (independent variables) with child care practices (dependent variable) (*n* = 272).

Independant Variables	Correrlation Coefficient
***Nutritional status of children***	
Stunted	0.0319
Wasted	−0.0028
Underweight	−0.0611
HAZ	0.0932 **
WAZ	0.0947 **
WHZ	0.0635
***Child’s factors***	
Average age of the child (months)	−0.5372 **
Child’s sex: boy	−0.0175
Average birth weight (kg)	0.1122 **
***Child’s health status in prior 2 weeks***	
Diarrhoea	−0.0542
Cough and cold	−0.0150
Fever	−0.0011
Average years of mother’s education	0.1355 **
Residency: urban	0.0296
Wealth Index: poorest	0.1479 **
Migrant status: father abroad	−0.2387 **

** Significant at the 5% level.

Table 4 presents the association of child, household variables, care practices and migrant status with the nutritional status of the child using the probit model. The area under the receiver operating characteristic curve (not presented here) is found to be 0.6596, 0.722, 0.7228 for stunting, wasting and underweight respectively, indicating that the estimated probit model fits efficiently. When the coefficient estimates of birthweight and wealth decrease stunting was observed to increase while stunting was seen to decrease with the increase in caregiving practices and when the father was a migrant worker. Birthweight and diarrhea have a negative association on wasting. Birthweight and poorest wealth have a negative association with underweight. The marginal effects highlight that, for each additional 100 gram increase in birthweight of children in the migrant household, the probability of being wasted, stunted and underweight decreased by 6, 8 and 24 percentage points respectively. Marginal effects of father being a migrant worker and improved childcare practices show that the likelihood of being stunted will be 0.11 and 0.02 percentage points lower. Considering the marginal effects it shows that the child having diarrhea during the past 2 weeks increases the probability of being wasted by 0.18 percentage points.

**Table 4:** Association of child, household variables and care practices by nutritional status—probit model estimation (*n* = 272).

Child and Household Variables	Estimate	Robust SE	Marginal Effect (In Percentages)
***Stunting***			
Constant	−1.2539	1.4285	
Birthweight	−0.3661 *	0.2241	−0.0613
Had diarrhoea	−0.4056	0.3802	−0.0679
Average caring practices	0.1313 *	0.0795	0.0220
Urban sector	0.3573	0.3432	0.0598
Average number of household members	−0.0917	0.0766	−0.0153
Poorest wealth	−0.1599 *	0.0893	−0.0268
Migrant father	0.6860 **	0.2739	0.1150
Log liklihood		−87.6	
***Wasting***			
Constant	1.7759	0.9939	
Average birthweight	−0.3649 *	0.2270	−0.0865
Had diarrhoea	−0.7778 **	0.3174	−0.1845
Average education of mother	−0.0450	0.0309	−0.0106
Log liklihood		−114.9	
***Underweight***			
Constant	1.3010	0.7204	
Average birthweight	−0.8005 ***	0.2073	−0.2303
Had cough and cold	0.2430	0.1230	0.0699
Being a girl	0.1738	0.1355	0.0500
Poorest wealth	−0.1883 **	0.0700	−0.0541
Log liklihood		−130.3	

*** Significant at the 1% level, ** significant at the 5% level, * significant at the 10% level.

## 4. Discussion

This study, aimed to explore the nutritional status and childcare practices of children left-behind in migrant households in Sri Lanka. Less than 5% of the study population had a parent who had migrated abroad for labour. Among those, in four out of five cases it was the father who was based abroad as a migrant worker. National labour migration statistics for 2014 indicated that of migrants working abroad, 63% are males and 37% are females [8].

Our study sample does not correlate to this ratio and the mean age of the child was higher in the migrant sample. This may be due to our study using the data from the national nutritional survey among children between 6–59 months and due to the law in Sri Lanka to ban international migration of women who have children younger than 3 years.

Our study found the lower level of mean z scores (HAZ, WHZ and WAZ) and lower prevalence of wasting, stunting and underweight among children in migrant households than in the non-migrant children and the overall sample.

Mixed results were reported from available studies. In Tonga, HAZ was lower among children younger than 18 years old left behind by migrants to New Zealand but no impact on WAZ. There was a positive impact on HAZ scores among Guatemalan children left behind by immigrants to the United States. Evidence from Tajikistan shows that children in communities with more migrants have higher HAZ-scores [7]. A 2006 survey conducted in migrant households of rural settings in selected areas of Pakistan suggests that migration is positively associated with the weight and height of both boys and girls, moreover among young children, the height was only significant for girls [24]. Evidence from a study in Tajikistan (2011) [25] suggests that the increase in household income due to remittances reduces malnutrition among children. Another study highlighted that the absence of a parent in a migrant household has a negative association on weight-for-height [26]. A longitudinal study in Mexico revealed that parental migration has a negative association on height-for-age [27]. It revealed that differences in data, country contexts, child age definitions, empirical specifications, and methods all may have contributed to the differences in these results, but they also highlight the complex relationship between migration and child nutrition, indicating further investigation [6].

Results revealed that childcare practices would decrease with younger children and when the father is a migrant worker. This may be associated with the limited time devoted to childcare practices by the remaining parent or caretaker [7]. This information is to be considered in developing policies on migrant workers. In addition childcare practices will increase with birth weight and wealth index suggesting that an increased household income may result in better improvements in socioeconomic status [28]. Our study shows that there is a positive relationship between mothers’ educational attainment and their children’s nutritional status. Higher level of education shows low wasting level of children. Previous studies have shown a similar observation [29].

Results from our analysis showed that stunting of children was significantly associated with wealth index of the migrant families. Proxy indicators of socioeconomic status used in the analysis and categorization of the wealth index was housing status and availability of electricity, television, refrigerator, mobile phones, sewing machine, radio and clock. Lower levels of stunting are seen in children with higher birthweight, better child care practices and a migrant father. Higher levels of stunting are seen in poorest households. Results from our analysis further highlight the need of better public policies towards migrant children. Although migrant workers migrate from their impoverished communities to improve socioeconomic status of their families, their children´s inadequate nutrition is an indicator of poor socioeconomic attainment. This needs further analysis.

Growth impairment during childhood has several consequences on physical and cognitive development. These consequences include decreased school performance and overall productivity, in addition to being a risk factor for chronic diseases later in life [10]. Migrant children living in poverty will have similar labour and productivity outcomes as their parents, thereby perpetuating the poverty and malnutrition cycle. Moreover, children with stunting, poor performance and scarce education opportunities, will have low productivity and poorer overall health as future adults [30].

Child’s birthweight is an important factor which affects nutritional status. This study shows that there is a positive relationship between birthweight and the child’s nutritional status, childcare practices and father being a migrant worker. Higher birthweight was associated with a low wasting, stunting and underweight level among children. Increase of 100 g of birthweight will decrease underweight by 23% in children of migrant households. A cross sectional study from Mexico identified that migration positively affects 364 g higher birthweight, and lowers the probability of children being underweight by 6.9%, controlling for other factors [31]. There is a known interaction between maternal height and birthweight and it is a known fact that breaking this intergenerational cycle will help to improve nutrition of children.

One of the goals of the migration as a determinant of development is to interrupt the intergenerational cycle of poverty by favouring the development of human capital, and by providing economic incentives for families to invest in their own future through education, health, and nutrition. However, this study highlights the importance of focusing on these issues developing favourable polices for children left-behind in migrant households [32].

The limitations to this study need to be considered. In the national survey, occupation of fathers and mothers of the children were collected during the last year. Although children 6–59 and 6–23 months of age have different care practices, analyses was done for 6–59 months due to low sample (*n* = 78) of 6–23 months even though the global norm of feeding practices for children 6–59 months of age is not available. The mother’s/caregiver’s reported personal hygiene and feeding practices were determined based on self-reported data as opposed to actual practice and may lead to problems in validity. Important variables to consider such as the number of children under five in the household, maternal height, duration of migration, internally migrated households and remittances were not included in the analysis, as the information was not available in the national survey. Despite these limitations, assessing the effects on nutritional status and care practices of children left-behind using nationally representative survey data in Sri Lanka is a critical step in strengthening the relevant evidence base and developing appropriate interventions for optimal child growth.

## 5. Conclusions

It appears that poor care giving practices and undernutrition that is commonly associated with poverty remains a major concern for child welfare in migrant families. Results from our analysis highlight the need for targeted nutritional programs towards potentially vulnerable and at risk children left-behind from migrant households, particularly from the poorest strata where the mother is the overseas based migrant worker and for low birth weight children. There is a need to build skills, capacities and preparedness for migrant families not only in terms of care provision for children left-behind but in better utilizing and investing remittances for poverty alleviation. The nutritional status of children left-behind may be influenced by a complex interplay of underlying social determinants and cultural gradients that extend beyond the effects of enhancing purchasing power for food due to remittance income, child care demands and food-preparation dynamics at the household level. Key areas to improve nutritional status of children are actions of nutritional surveillance and nutrition promotion among migrant families. Further representative and in depth studies are needed in the future to explore more factors associated with nutritional status of children left-behind in migrant families and how labour migration affect household level outcomes related to poverty, child nutrition and child care.
